# Thrombophilias and Pregnancy Complications: A Case-Control Study

**Published:** 2007-09

**Authors:** Larciprete Giovanni, Angelucci Piero Antonio, Celleno Danilo, Gioia Stefano, Deaibess Therese, Romanini Maria Elisabetta, Brienza Letizia, Cirese Elio, Arduini Domenico

**Affiliations:** 1 *AFaR, Associazione Fatebenefratelli per la Ricerca, Ospedale Fatebenefratelli Isola Tiberina, Rome, Italy;*; 2 *Department of Perinatal Medicine, Tor Vergata University, Rome, Italy;*; 3 *UOC Anestesia e Rianimazione, Ospedale Fatebenefratelli Isola Tiberina, Roma;*; 4 *Clinica Ostetrica, Policlinico Umberto I, Università la Sapienza, Roma*

**Keywords:** thrombophilias, pregnancy, Antithrombin III deficiency, preeclampsia, disseminated intravascular coagulation

## Abstract

Inherited thrombophilia is believed to be a multiple gene disease with more than one defect. We wanted to determine the association between single thrombophilic patterns and a variety of pregnancy diseases. 301 pregnant women were recruited for the present case-control study and were divided into two groups: A group (176 controls) and B group (125 cases). Patients belonging to the B group had one of the following: severe preeclampsia, HELLP syndrome, gestational hypertension, fetal growth restriction (FGR), intrauterine death, abruptio placentae, placenta previa, disseminated intravascular coagulopathy (DIC) and preterm labour. To detect MTHFR A1298C, MTHFR C677T, Factor V Leiden, PAI-1, Mutant Prothrombin G20210A, an inverse hybridization technology was used. Plasma homocysteine, Antithrombin III and protein levels S were determined. A modified functional activated protein C resistance was assayed. MTHFR C677T and hyperhomocysteinemia were more numerous than other thrombophilias. Deficiency in AT III was significantly linked with preeclampsia (Pearson Index and p value: 0.131 and 0.022, respectively) and disseminated intravascular coagulopathy (Pearson Index and p value: 0.138 and 0.016 respectively). Activated Protein C resistance was related to abruptio placentae (Pearson Index and p value: 0.159 and 0.005 respectively). Apart from the linkage between AT III deficiency and the occurrence of preeclampsia and disseminated intravascular coagulopathy, we obtained findings in contrast to some literature. In our case series, no association of preeclampsia with Factor V Leiden or with prothrombin gene mutation was found.

## INTRODUCTION

Inherited thrombophilia is believed to be a multiple gene disease with more than one defect, which explains why some women with thrombophilia never have a thrombotic event, whereas others have complications. This condition is generated by specific point mutations (single-nucleotide polymorphism) including factor V Leiden mutation (G1691A Factor V), methylenetetrahydrofolate reductase (MTHFR) mutations (C677T MTHFR and A1298C MTHFR), G20210A prothrombin (G20210A PTR) gene mutation, and plasminogen activator inhibitor-1 mutant genotype (PAI-1 5G/5G). Other thrombophilias incude deficiencies in antithrombin III (AT III), protein S, protein C, resistance to the activated protein C (APCR) and elevated homocysteinemia (1).

Thrombophilias have been recently explored as cause a of placental thrombosis, severe preeclampsia/eclampsia, HELLP syndrome, placental abruption, intrauterine growth restriction, unexplained stillbirth and recurrent miscarriage (2).

The link between thrombophilia and certain pathologies of pregnancy may be an inadequate feto-placental circulation (3).

Kupferminc *et al*. (3), showed a further evidence of inherited and acquired thrombophilias in relation to adverse pregnancy outcome, by means of a study on Israeli women with serious pregnancy complications compared with healthy pregnant women.

## AIM OF THE STUDY

The aim of the present study was to determine to what extent single inherited thrombophilias are associated with adverse obstetric complications correlated with uteroplacental insufficiency such as preeclampsia, HELLP syndrome, gestational hypertension, fetal growth restriction (FGR), intrauterine death, abruptio placentae, placenta previa, disseminated intravascular coagulopathy (DIC) and preterm labour.

## METHODS

### Patients’ selection

Since September 2002 until May 2005, pregnant women coming to our Obst/Gyn tertiary care unit of Fatebenefratelli Isola Tiberina (Rome) were enrolled consecutively in the present prospective case-control study, at the entrance. All these women delivered at the Department of Obstetrics and Gynecology of Fatebenefratelli Hospital, Isola Tiberina, Rome. Patients were scheduled in two study groups, according to the absence (Group A, Controls) or presence (Group B, Cases) of any of the cited adverse pregnancy outcomes correlated with uteroplacental insufficiency such as severe preeclampsia, HELLP syndrome, gestational hypertension, fetal growth restriction (FGR), intrauterine death, abruptio placentae, placenta previa, disseminated intravascular coagulopathy (DIC) and preterm labour.

Inclusion criteria to be recruited within the Group B were: HELLP syndrome, defined as severe preeclampsia complicated with hemolysis (total bilirubin >1.2 mg/dl, lactic dehydrogenase >600 U/l), hepatic enzymes increase (aspartate aminotransferase >70 U/l) and thrombocytopenia (platelets count <100,000/mm^3^) (2); fetal growth restriction (FGR), defined as neonatal weight <10° percentile (2); placental abruption, assessed clinically on the basis of antepartum uterine tenderness and vaginal bleeding and confirmed by inspection of the placenta at delivery (2); intrauterine death (ID), defined as the delivery of a dead fetus after 24 weeks of gestation (2); gestational hypertension (GH), defined as an increase of systolic blood pressure of 30 mmHg or higher and/or increase of diastolic blood pressure of 15 mmHg or higher from average values before 20 weeks’ gestation (In case of unknown prior values, two readings of 140/90 mmHg or higher on two different occasions more than 4 hours apart (4)); preterm labour (PL), defined as delivery before 37 weeks’ gestation; placenta previa, defined as the chorionic plate lying just upon the inner uterine orifice after 30 weeks’ gestation (ultrasound transvaginal scan); disseminated intra-vascular coagulopathy (DIC), was defined according to Levi M *et al* (5); definition of preeclampsia was based on recommendations of the Consensus Report of the American Working Group on High Blood Pressure in Pregnancy and of the Working Group of the German Society of Obstetrics and Gynecology (4). Diagnostic criteria were as follows:

- Increase of systolic blood pressure of 30 mm Hg or higher and/or increase of diastolic blood pressure of 15 mm Hg or higher from average values before 20 weeks’ gestation. In case of unknown prior values, two readings of 140/90 mm Hg or higher on two different occasions more than 4 hours apart, is considered diagnostic of preeclampsia.
- Proteinuria is defined as protein excretion of 0.3 g or more in a 24 hour specimen or repeated dipsticks of 30 mg/dL (which correlates to 1 + dipsticks in commercial kits) in two random urine specimens collected more than 4 hours apart.
- Onset of first symptoms beyond 20 weeks’ gestation from last menstrual period and regression of symptoms after 6 weeks postpartum.
- Absence of pre-existent hypertension, proteinuria, and edema, as well as diabetes, chronic kidney, hepatic, or vascular disease.

Pregnancies with fetal congenital anomalies and women with chronic hypertension, diabetes mellitus and pre-existing renal disease were excluded from the recruitment. A peripheral venous blood sample was taken from every recruited case. Women with combined thrombophilic defects were excluded from the recruitment.

Patients with MTHFR (any type) mutant genotype (homo/heterozygous) or hyperhomocysteinemia (more than 12 mol/L) were invariably instructed to ingest folates daily until delivery (6), following an internal departmental treatment protocol.

### Laboratory assay

To detect MTHFR A1298C, MTHFR C677T, Factor V Leiden, PAI-1, Mutant Prothrombin G20210A, a commercially available kit was employed following the manufacturer procedures (FV-Protrombina, MTHFR C677T/A1298C, HPA1a/b, APOB; Nuclear Laser Medicine, AC007, Milan, Italy).

Briefly, an inverse hybridization technology is used: DNA from EDTA blood samples (maintained at –20°C) was isolated after lysis by means of a GenXtract resin, as reported in the kit instructions. A subsequent simultaneous DNA amplification (multiplex) by Taq polymerase and amplification mix was obtained with 35 cycles in thermocycler (GeneAmp PCR System 2400 of the Perkin Elmer, Milan, Italy).

The amplified fragment was hybridized on a membrane with different allele-specific probes linked to Biotin. Biotin is subsequently revealed by streptavidine-conjugated alkaline phosphatase exposed to appropriate coloured substrate. This colorimetric reaction allows the detection of mutations.

Plasma homocysteine determinations were carried out using a commercially available kit (Axis Homocysteine EIA, Axis-Shield Diagnostics Ltd, The Technology Park, and Dundee DD2 1Xa, UK) and following the manufacturer’s instructions: Intra-CV and inter-CV were less than 6% and 12%, respectively. The normal reference range is 5-12 μmol/L and the sensitivity is 1 μmol/L.

Antithrombin III, amydolytic and immunologic (Behring, Marburg, Germany) and total and free (ELISA; Diagnostica Stago, Asniéres, France) protein S antigen were determined in all subjects, as reported elsewhere (7, 8).

Inter- and intra-assay coefficients of all the variables never exceeded 8.0 and 5.0%, respectively.

A modified functional activated protein C resistance was detected using factor V-deficient plasma (Coatest activated protein C resistance-V; Chromogenix; Goteborg, Sweden), as previously described (9).

Informed consent to utilize the data for this work was obtained by the outpatients included in this study. The study was approved by the ethical committee of the Fatebenefratelli Hospital.

### Statistical analysis

For a power of 95% and a type I error of 0.01 we needed 100 patients per group, and, for a power of 90% and a Type I error of 0.001 we needed 110 patients (10). Homozygous mutations were taken into consideration for final conclusions.

Student T test was used to assess differences between groups and Pearson correlation 2-tailed method was used to check correlations between thrombophilic patterns and the occurrence of adverse pregnancy outcomes.

## RESULTS

Characteristics of the study groups are summarized in Table 1. There were no differences between the two groups regarding maternal age and pregravidic BMI (Body Mass Index), but significant differences were noted about the gestational age at delivery (39 ± 2 weeks vs. 35 ± 4 weeks, *p*=0.045), the birthweights (3325 ± 398 g vs. 2560 ± 256 g, *p*=0.034) and the gestational weeks at the enrollement (39 ± 2 weeks vs. 26 ± 8 weeks, *p*=0.024).

**Table 1:** Characteristics of the study groups. Student t-test comparison

	Group A	Group B	p-values
	n. 176	n. 125	

Age (years)	33.2 ± 5.1	34.7 ± 4.1	0.124
Pre-pregnancy BMI	24.4 ± 3.1	25.0 ± 3.3	0.235
G.A. at delivery (weeks)	39 ± 2	35 ± 4	0.045
Birthweight (g)	3325 ± 398	2560 ± 256	0.034
G.A. at enrollment (weeks)	39 ± 2	26 ± 8	0.024

G.A., gestational age.

Three hundred eighteen pregnant women were enrolled in the present study. Seventeen out of them were excluded: 10 had double or triple thrombophilic patterns, 3 had chronic hypertension, 1 patient had a fetus with congenital cardiac abnormalities, 3 patients had type 1 diabetes mellitus. Therefore, the 301 subjects enrolled for the final study were divided into two study groups according to the absence (Group A, 176 subjects) or presence (Group B, 125 subjects) of any of the cited adverse pregnancy outcomes correlated with uteroplacental insufficiency that are preeclampsia, HELLP syndrome, gestational hypertension, fetal growth restriction (FGR), intrauterine death, abruptio placentae, placenta previa, disseminated intravascular coagulopathy (DIC) and preterm labour. Pregnancy outcomes are described in Table 2, being the fetal growth restriction the most representative adverse pregnancy outcome of our series.

**Table 2:** Distribution of the normal and pathological pregnancy outcomes

Outcome	N.	Frequency (%)

Normal pregnancies	176	58.80
Intrauterine death	5	1.64
Gestational hypertension	25	8.22
Preeclampsia	7	2.30
HELLP syndrome	14	4.60
Fetal Growth Restriction	39	12.82
Abruptio placentae	16	5.26
Previa	10	3.28
DIC	2	0.65
Preterm labour	7	2.43

In both study groups the occurrence of the single thrombophilic genotypes had a wide variation and the incidence of MTHFR C677T heterozygous or homozygous pattern and the appearance of hyperhomocysteinemia were greater than that observed for the other thrombophilic subtypes (Table 3). In our series we didn’t observe cases with either MTHFR mutations combined with hyperhomocysteinemia.

**Table 3:** Distribution of the thrombophilic patterns

Outcome	N.	Frequency (%)

AT III deficiency	12	4.15
Protein S deficiency	1	0.34
APCR	53	18.33
Hyper-HCY	20	6.92
MTHFR C677T		
+/-	91	31.48
+/+	69	23.87
MTHFR A1298C		
+/-	2	0.69
+/+	5	1.73
G20210A PTR		
+/-	1	0.34
+/+	2	0.69
G1691A factor V		
+/-	14	4.84
+/+	2	0.69
PAI-1 (5G/5G)		
+/-	10	3.46
+/+	7	2.42

AT III, antithrombin III; PAI, plasminogen activator inhibitor; MTHFR, methylenetetrahydrofolate reductase; G20210A PTR, single-nucleotide polymorphism of prothrombin (PTR); G1691A factor V, factor V Leiden; hyper-HCY, hyperhomocysteinemia; APCR, activated protein C resistance.

Correlations between thrombophilic patterns and adverse pregnancy outcomes are summarized in Table 4, showing, per each adverse pregnancy outcome, the number of affected subjects with a single thrombophilic mutation, with the relative Pearson correlation coefficient and significance.

**Table 4:** Correlations between thrombophilic patterns and adverse pregnancy outcomes

Outcome	*ID*	*GH*	*PE*	*HEELP*	*FGR*	*PL*	*Abruptio*	*Previa*	*DIC*

	Cases with single mutation Pearson correlation 2-tailed significance								

AT III deficiency	0	2	6	0	2	0	0	0	2
	-0.035	-0.034	0.131	0.002	0.012	-0.042	0.052	-0.050	0.138
	0.541	0.551	0.022	0.972	0.837	0.468	0.366	0.383	0.016
Protein S deficiency	0	0	0	0	1	0	0	0	0
	-0.024	-0.055	-0.028	-0.041	-0.016	-0.028	-0.043	-0.034	-0.015
	0.679	0.337	0.623	0.481	0.786	0.623	0.450	0.555	0.794
APCR	1	4	0	3	12	3	11	4	0
	-0.028	-0.066	-0.034	-0.048	0.010	-0.034	0.159	-0.041	-0.018
	0.622	0.253	0.558	0.402	0.868	0.558	0.005	0.481	0.756
Hyper-HCY	0	3	0	2	6	0	2	2	0
	0.046	-0.016	-0.050	0.089	0.110	-0.050	-0.026	-0.060	-0.026
	0.424	0.785	0.386	0.122	0.056	0.386	0.647	0.298	0.122
C677T MTHFR (+/+)	1	4	1	2	9	2	3	4	0
	-0.061	0.039	-0.084	-0.041	0.131	-0.084	-0.023	-0.041	0.044
	0.286	0.493	0.144	0.478	0.022	0.144	0.695	0.477	0.448
A1298C MTHFR (+/+)	0	2	0	0	3	0	0	0	0
	-0.032	-0.041	-0.038	0.033	0.070	0.146	-0.017	-0.046	-0.020
	0.576	0.476	0.506	0.563	0.224	0.011	0.762	0.425	0.725
G20210A PTR (+/+)	0	0	0	1	1	0	0	0	0
	-0.027	-0.063	-0.032	0.016	-0.002	-0.032	0.009	0.108	-0.017
	0.638	0.275	0.576	0.775	0.971	0.576	0.872	0.059	0.767
G1691A factor V (+/+)	0	1	0	0	1	0	0	0	0
	-0.038	-0.089	-0.046	-0.065	0.029	-0.046	0.102	-0.001	-0.024
	0.505	0.122	0.428	0.257	0.611	0.428	0.077	0.985	0.675
PAI-1 (5G/5G) (+/+)	1	3	0	1	2	0	0	0	0
	0.005	-0.035	-0.054	-0.016	0.018	-0.054	-0.054	0.007	-0.029
	0.934	0.54	0.350	0.779	0.755	0.350	0.348	0.906	0.620

AT III, antithrombin III; PAI, plasminogen activator inhibitor; MTHFR, methylenetetrahydrofolate reductase. APCR, activated C-protein Resistance; G20210A PTR, single-nucleotide polymorphism of prothrombin (PTR); G1691A factor V, factor V Leiden; hyper-HCY, hyperhomocysteinemia.

Deficiency in AT III was found to be significantly linked with the occurrence of preeclampsia (Six of seven cases with ATIII deficiency, Pearson Index and p value: 0.131 and 0.022, respectively) and disseminated intravascular coagulopathy (Two of two cases with ATIII deficiency, Pearson Index and p value: 0.138 and 0.016, respectively). Activated Protein C resistance was found to be strictly related to the abruptio placentae (Eleven of sixteen cases with Activated protein C Resistance, Pearson Index and p value: 0.159 and 0.005, respectively).

We didn’t find an increased incidence of adverse pregnancy outcomes in subjects with protein S deficiency, hyperhomocysteinemia, C677T or A1298C MTHFR homozygous mutation, G20210A Prothrombin homozygous mutation, G1691A factor V homozygous mutation, PAI-1 homozygous mutation.

## COMMENTS

Since preeclampsia is associated with vascular and endothelial damage, which in turn is linked to coagulation problems, congenital thrombophilias may play an important role in this pathology.

For blood fluidity and wall repair, the placental vessels, like those at other sites, should maintain equilibrium between the procoagulant and anticoagulant mechanisms: an imbalance may lead to placental infarction on one or both of the placental sides (11).

The factor V Leiden mutation results from a substitution of adenine for the normal guanine at the 1691 position of the factor V gene. As a result, factor V becomes resistant to cleavage by activated protein C. The factor V heterozygote is present in approximately 5.2% of white Americans and in 1.2% of African-Americans (12). In our series it was not linked to any adverse pregnancy outcome, as a single homozygous gene mutation.

Protein S deficiency is an autosomal dominant disorder that exposes the fetus to an increased risk of thromboembolism. Protein S is a Vitamin K-dependent plasma protein cofactor that is necessary along with activated protein C cofactor to inactivate factors Va and VIIIa and control the balance between coagulation and anticoagulation. Sanson *et al*. (13) showed that the relative risk of abortion and stillbirth per pregnancy in women with protein S, protein C, and antithrombin deficiencies was 2.0 times greater (95% CI 1.2–3.3) than in non-deficient women. Again, in our series, the S protein deficiency did not lead to any of the studied adverse pregnancy outcomes, with the exclusion of abortion, not studied in this case-series.

Another significant cause of thrombophilia in pregnancy is activated protein C resistance (APCR) (14). Inherited APCR is an autosomal dominant disorder and is one of the most common forms of inherited thrombophilic disorders, with a prevalence of 5% in the general population (15, 16). In the majority of cases, APCR is due to a point mutation in the factor V gene, which prevents protein C from inactivating active factor V (14). In our series this thrombophilia had a frequency of 18.3% and was significantly linked to the abruptio placentae, being present in 10 of 16 cases.

Inherited hyperhomocystinemia is another cause of thrombophilia that results from genetic defects in methionine and homocysteine metabolism, which leads to recurrent venous thrombosis. The human methylenetetrahydrofolate reductase (MTHFR) gene, which is located on chromosome 1p36, belongs to the proposed candidate loci for preeclampsia (17). The MTHFR gene is critical in the metabolism of homocysteine because the reaction catalyzed by MTHFR is a rate-limiting step in the folate cycle and can be affected by an individual’s folate status.

A common missense mutation at nucleotide 677, which substitutes a valine for an alanine residue, has been associated with increased circulating levels of homocysteine caused by decreased enzyme activity in C677T homozygotes and heterozygotes (18). Hyperhomocystinemia can induce vascular injury, increased platelet consumption, and can result in thrombosis caused by increased oxidative stress. Clinically, hyperhomocystinemia caused by the C677T mutation has been implicated in premature cardiovascular disease (19), venous thrombosis (20), and more recently in adverse pregnancy outcome, especially preeclampsia (17). However, the majority of follow-up studies failed to reconfirm a significant disease association with preeclampsia (21, 22, 23). Because the frequency of the C677T allele underlies significant population-specific differences, it was proposed that variations in the relative contribution of disease alleles in different populations might explain the discrepant results of previous studies on MTHFR and preeclampsia (24). This could explain why in our series we didn’t observe any significant association between MTHFR homozygous mutations and adverse pregnancy outcomes, despite the high incidence of this thrombophilias in our study population.

Recently, a second common mutation in the MTHFR gene has been described, an adenine-to-cytosine substitution at base 1298 (A1298C). This mutation also results in decreased MTHFR activity but it is not associated with higher plasma homocysteine concentration or lower plasma folate concentration (25).

Hernandez-Diaz (26) and Glanville (27) have recently showed a tight linkage between MTHFR mutations, hyperhomocysteinemia and both preeclampsia and intrauterine growth restriction.

In our series nor the C677T MTHFR neither the A1298C MTHFR was associated with adverse pregnancy outcomes. Apart from the above cited considerations (24), we also speculate that oral folate supplementation could decrease the incidence of both hyperhomocysteinemia and adverse pregnancy outcomes in these patients.

Plasminogen activators (PA) induce changes in the fibrinolytic system that convert plasminogen to plasmin. During pregnancy, the anticlotting activity of PA is kept in check by two plasminogen activator inhibitors, one of which (PAI-1) is endothelial cell related and the other (PAI-2) is produced in placental tissue (28).

The concentrations of both PAI-1 and PAI-2 increase during pregnancy (29) in order to ensure hemostasis during labor and delivery. The mutant PAI-1 5G/5G homozygous genotype may be involved in the development of intrauterine growth restriction (IUGR) as well as preeclampsia (30, 31). Even in this case, we were unable to detect any association between this thrombophilic pattern and adverse pregnancy outcomes.

Presumably, a similar increase in coagulation may be responsible for the increased incidence of miscarriage and fetal demise seen in protein C, S and ATIII deficient patients (32, 33).

Gerhardt *et al*. presented a case-control study of 97 women with severe preeclampsia in previous pregnancies and 277 normal women, to assess hereditary risk factors of venous thrombosis as risk determinants for severe preeclampsia. In his research, the onset of severe preeclampsia was significantly earlier in women with the G20210A prothrombin gene mutation (24.5 weeks vs. 30.1 weeks, *P*=0.046) and in women with the PAI-15G-5G genotype (25.7 weeks vs. 30.8 weeks, *P*=0.024), showing that these risk factors do not induce the pathomechanism but accelerate the course of preeclampsia (34).

In a recent prevalence study on 200 patients, the G20210A single-nucleotide polymorphism of prothrombin was shown to be unrelated to recurrent spontaneous abortions, whereas factor V Leiden, along with APCR, as well as combination of both, were seen in women with idiopathic recurrent pregnancy loss, suggesting a close linkage with this pathology.

Apart from the linkage between AT III deficiency and the occurrence of preeclampsia and disseminated intravascular coagulopathy, we obtained findings in contrast with Gerhardt A *et al*. In our case series, we had no association between preeclampsia and Factor V Leiden or prothrombin gene mutation. The mechanism of impact of single inherited thrombophilias on abruptio placentae surely deals with microangiopathy, a common pattern found within the thrombophilic population, but it is far to be demonstrated.

Our study deals with single thrombophilias and is poorly comparable with others. Moreover, it is important to underline that in our study we had no subject with recurrent spontaneous miscarriages because our study population was enrolled within a tertiary care unit and not within an outpatient clinic.

We enrolled subjects beyond the statistically recommended numbers necessary to reach strong evidence.

The main result of our research was that, excluding multiple gene mutation, single thrombophilias have to be carefully taken into consideration, but without generating unjustified fears about pregnancy.

Further studies are needed to check the linkage between thrombophilic gene mutations and adverse pregnancy outcomes.
